# Yellow Fever Inoculation

**Published:** 1893-05

**Authors:** J. McF. Gaston


### YELLOW FEVER INOCULATION.

A recent report of Dr. Domingos Freire appeared in the *Medical News*, of Philadelphia, substantiating his former good results from yellow fever inoculation. This was accompanied with a brief communication from me, to which Dr. George M. Sternberg has replied in the *News*.

In making a criticism upon his mode of dealing with Freire's statistics, if plain, direct expression of my convictions cannot be accepted, there is no prospect of reaching a correct conclusion of this issue, and my only aim in refering to this matter is that the medical profession may know how difficult it is to get a hearing, when in antagonism with representatives of the medical department of the Army of the United States.

The communication which the *News* has published, is as follows :

" In reply to Dr. George M. Sternberg, in your issue of April 8th, I have only to draw the attention of your readers to a few points. Referring to the year 1885, Dr. Sternberg says, 'Dr. Freire has included in his list 1,294 persons who were vaccinated during the healthy winter months of June and July and who were exposed during the preceding comparatively unhealthy months of January, February, March and April.' If these 1,294 individuals were protected from an attack of yellow fever by the inoculation practiced in June and July, what protected them from being attacked during the preceding epidemic season ?

" This interrogatory loses all its point when Dr. Sternberg is reminded of his own figures, showing that in the entire population of Rio de Janeiro, there were only 278 deaths from yellow fever during the year of 1885, the early part of which he refers as the preceding epidemic season. Readers should take into account this fact, and that in the subsequent year, 1886, there were 1,397 deaths from yellow fever in that city.

"Looking further into the matter, he says 'it is a significant fact that, of the 3,051 vaccinated prior to August, 1885, Dr. Freire has only one fatal case to report, while out of 460 vaccinated in January and February, 1886, he reports five deaths, a mortality of more than one per cent, which he gives as general mortality among the non-vaccinated.'

"That is to say, the mortality among those vaccinated during those two epidemic months, was more than one per cent. On referring to the mortality of the city for the same two months, says Dr. Sternberg, 'I find the total number of deaths to have been 369, which, in a total susceptible population of 160,000, (Dr. Freire's estimate,) would give a mortality of one in 436.' That figures do not lie, may be true, when a calculation is correctly made, but Dr. Sternberg may be excused for slight errors in his statement as given in the last figures above. In qualifying the above statement he goes on to remark: 'That is, the mortality among those vaccinated during those two months of epidemic prevalence of yellow fever was very much greater than among the non-vaccinated portion of the population.'

"This is the climax of Dr. Sternberg's array of figures against the statistics of Freire, that more of his inoculated cases died in a given period than the proportion of deaths from all causes amongst the population of the entire city, including, of course, in the general result, these identical cases laid at the door of Domingos Freire.

"Unless it is alleged in the declaration of charges against the process of inoculation adopted by Freire that it actually increased the mortality of those subjected to it, we cannot accept the verdict brought in by Dr. Sternberg, that the mortality among those vaccinated during these two months of epidemic prevalence of yellow fever was very much greater than among the non-vaccinated portion of the population.

"It is in order to give a report in this connection of the experience of some of the missionaries from the United States in Brazil, as to the consequences of yellow fever inoculation. Five ministers of the gospel have died from yellow fever in Brazil within a few years, who, to say the least, failed to be inoculated, viz : Messrs. Koger, Thompson, Dabney, Pinkerton and E. Lane. On the other hand, Dr. H. M. Lane, was inoculated, and has been often exposed without suffering from yellow fever."

J. McF. GASTON, M. D.

